# Diketopyrrolopyrrole‐Based Conjugated Polymer Entailing Triethylene Glycols as Side Chains with High Thin‐Film Charge Mobility without Post‐Treatments

**DOI:** 10.1002/advs.201700048

**Published:** 2017-04-18

**Authors:** Si‐Fen Yang, Zi‐Tong Liu, Zheng‐Xu Cai, Matthew J. Dyson, Natalie Stingelin, Wei Chen, Hua‐Jun Ju, Guan‐Xin Zhang, De‐Qing Zhang

**Affiliations:** ^1^ Beijing National Laboratory for Molecular Sciences CAS Key Laboratory of Organic Solids Institute of Chemistry Chinese Academy of Sciences Beijing 100190 P. R. China; ^2^ University of Chinese Academy of Sciences Beijing 100049 P. R. China; ^3^ Department of Materials and Centre for Plastic Electronics Imperial College London London SW72AZ UK; ^4^ Materials Science Division Argonne National Laboratory 9700 Cass Avenue Lemont IL 60439 USA; ^5^ Institute for Molecular Engineering The University of Chicago 5640 South Ellis Avenue Chicago IL 60637 USA

**Keywords:** charge carrier mobility, conjugated donor–acceptor polymer, diketopyrrolopyrrole, side chains, triethylene glycol

## Abstract

Side chain engineering of conjugated donor–acceptor polymers is a new way to manipulate their optoelectronic properties. Two new diketopyrrolopyrrole (DPP)‐terthiophene‐based conjugated polymers PDPP3T‐1 and PDPP3T‐2, with both hydrophilic triethylene glycol (TEG) and hydrophobic alkyl chains, are reported. It is demonstrated that the incorporation of TEG chains has a significant effect on the interchain packing and thin‐film morphology with noticeable effect on charge transport. Polymer chains of PDPP3T‐1 in which TEG chains are uniformly distributed can self‐assemble spontaneously into a more ordered thin film. As a result, the thin film of PDPP3T‐1 exhibits high saturated hole mobility up to 2.6 cm^2^ V^−1^ s^−1^ without any post‐treatment. This is superior to those of PDPP3T with just alkyl chains and PDPP3T‐2. Moreover, the respective field effect transistors made of PDPP3T‐1 can be utilized for sensing ethanol vapor with high sensitivity (down to 100 ppb) and good selectivity.

## 1 Introduction

Conjugated electron donor–acceptor (D‐A) polymers have been intensively explored over the last decades in order to develop higher performing organic semiconductors and photovoltaic materials.^1^, ^2^, ^3^, ^4^, ^5^ They have been successfully utilized for fabrication of thin film transistors with high charge mobilities and photovoltaic cells with high power conversion efficiencies,^6^, ^7^, ^8^, ^9^, ^10^, ^11^, ^12^, ^13^, ^14^ clearly demonstrating the promise of conjugated D‐A polymers for use in solution‐processible, large‐area, low‐cost, and flexible electronic devices.^15^, ^16^ The semiconducting properties of this interesting “plastics” are primarily governed by the electronic structure of their conjugated backbones, along with interchain interactions.^17^ In this respect, various electron acceptors and donors have been devised and incorporated into the polymer backbones to tune their highest occupied molecular orbital (HOMO)/lowest unoccupied molecular orbital (LUMO) levels and backbone conformations as well as their molecular arrangement and packing.^4^, ^10^, ^18^ Thereby, thiophene, bithiophene, and the selenium analogs are widely employed as electron donors, whereas imide and diimide‐containing heterocyclic molecules such as diketopyrrolopyrrole (DPP), isoindigo, naphthalenediimide, and Pechmann dyes are chosen as electron acceptors.^19^, ^20^, ^21^, ^22^, ^23^, ^24^, ^25^, ^26^, ^27^

Recent studies demonstrate that the alkyl side chains in conjugated D‐A polymers not only improve solubility but also influence interchain packing and thus affect their semiconducting properties. For instance, it was reported that charge mobilities of isoindigo/DPP‐based conjugated polymers were enhanced by moving the branching points of branching alkyl chains away from the conjugated backbone.^2^, ^28^ Increased charge mobilities were also reported for conjugated polymers where pure alkyl side chains were replaced with siloxane‐terminated alkyl, fluoroalkyl, or oligo(ethylene glycol) chains.^29^, ^30^, ^31^, ^32^, ^33^, ^34^, ^35^, ^36^ Taking advantage of the formation of hydrogen bonding between urea groups, we introduced urea groups in the side alky chains of DPP‐quaterthiophene conjugated polymers to improve interchain packing order and thin‐film morphology, resulting in large enhancement of hole mobilities.^33^

In comparison with alkyl chains, triethylene glycol (TEG) is polar and hydrophilic. Therefore, it is expected that the incorporation of TEG into the side chains of conjugated polymers will alter the interactions among side chains and thus affect the packing and interactions among the conjugated backbones. In fact, Yang and co‐workers introduced TEG into the side chains of terpolymers as electron donors for photovoltaic cells with [6,6]‐phenyl‐C_71_‐butyric acid methyl ester (PC_71_BM).^34^ In these terpolymers some DPP units were connected to alkyl chains, while the TEG chains were linked to the rest of DPP units. The presence of TEG chains in this system resulted in the electron donors and acceptors to pack more regularly, affecting the respective domain structures and enhancing photovoltaic performance. Patil and co‐workers reported that a DPP–DPP polymer with TEG in the side chains exhibited high electron mobility up to 3.0 cm^2^ V^−1^ s^−1^ after thermal annealing.[[qv: 31a]] Nevertheless, investigation of conjugated D‐A polymers with TEG chains is limited and merits further exploration.

**Scheme 1:**
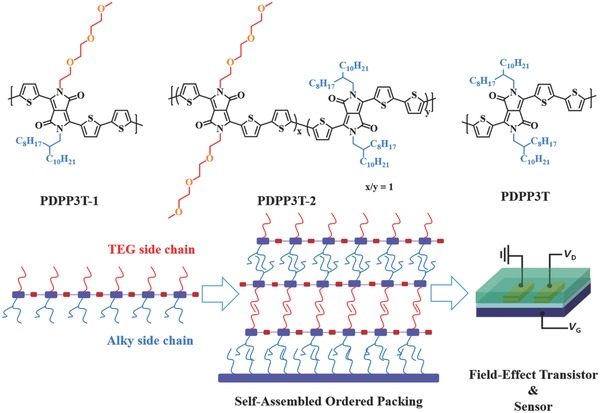
Chemical structures of PDPP3T‐1, PDPP3T‐2, and PDPP3T and illustration of the design rationale for incorporation of TEG side chains in the conjugated polymers.

In this paper, we report a new DPP‐terthiophene‐based conjugated polymer, PDPP3T‐1 (**Scheme** **1**), in which each DPP unit has both an alkyl and a TEG chain. To investigate the influence of TEG chains in PDPP3T‐1 on the interchain packing order and the semiconducting performance, the reference polymers PDPP3T and PDPP3T‐2 were also prepared. As depicted in Scheme 1, each DPP unit in PDPP3T is uniformly connected to two alkyl chains (2‐octyldodecane). For the terpolymer PDPP3T‐2, however, half of the DPP units have alkyl side chains while the remainder have TEG chains. We show here that (i) polymer chains of PDPP3T‐1 can self‐assemble spontaneously into more ordered thin films than PDPP3T and PDPP3T‐2 and (ii) as‐prepared thin films of PDPP3T‐1 exhibit high hole mobility up to 2.6 cm^2^ V^−1^ s^−1^ without any post‐treatments, whereas those of PDPP3T and PDPP3T‐2 possess low charge carrier mobilities under the same conditions. Therefore, it can be concluded that the influence of TEG chains on the semiconducting performance of conjugated D‐A polymers is governed by the arrangement of TEG chains along the conjugated backbone. In addition, field‐effect transistors with thin films of PDPP3T‐1 show sensitive and selective response to alcohol vapors by taking advantage of the presence of TEG chains in PDPP3T‐1.

## 2 Results and Discussion

**Scheme 2:**
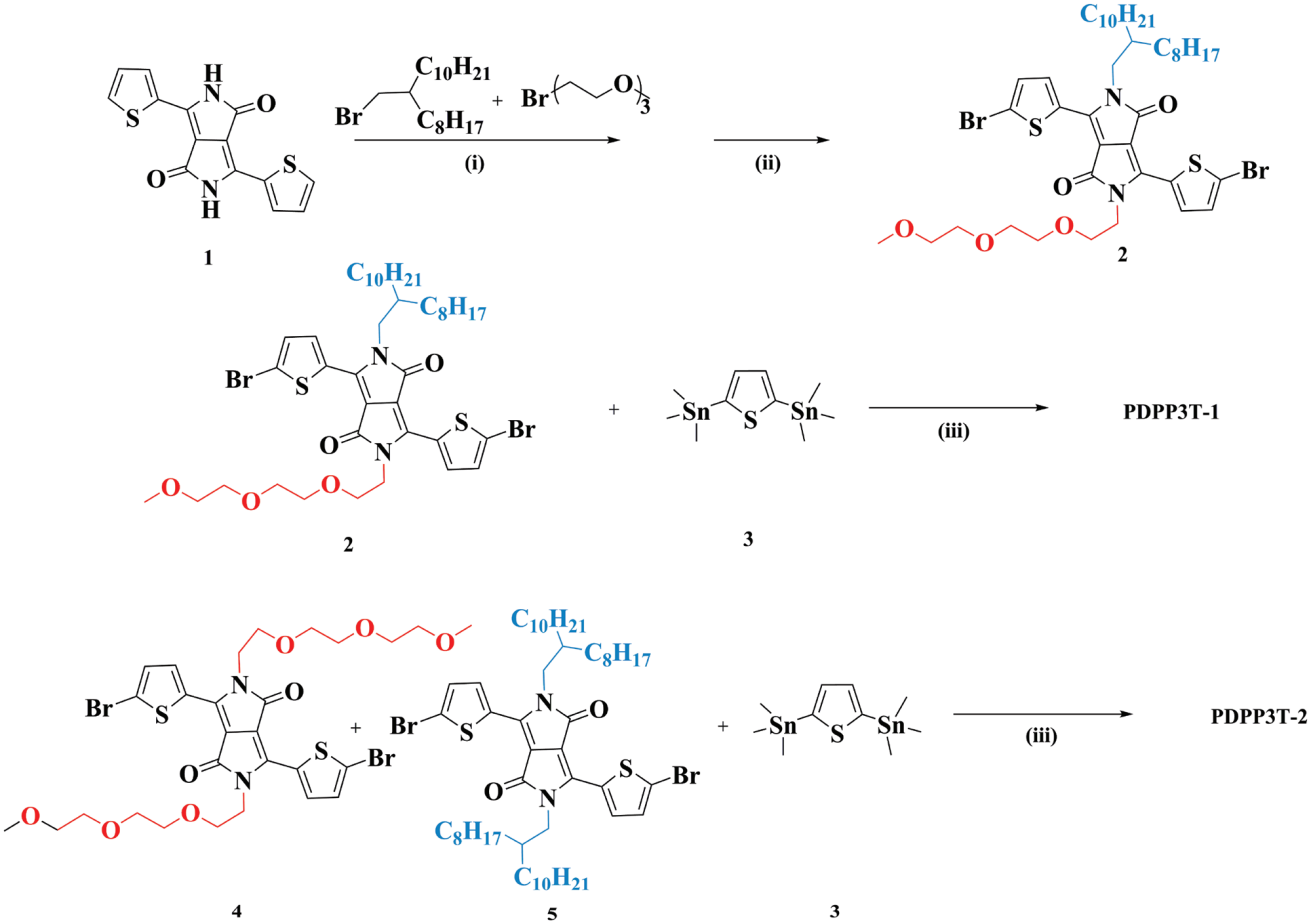
Synthetic routes to PDPP3T‐1 and PDPP3T‐2. Reagents and conditions: (i) K_2_CO_3_, N,N‐Dimethylformamide (DMF), 130 °C, 36 h; (ii) *N*‐bromosuccinimide, CHCl_3_, r.t., 4 h; (iii) Pd_2_(dba)_3_, P(*o*‐tol)_3_, toluene, 100 °C, 36 h.

The synthesis of PDPP3T‐1 and PDPP3T‐2 is outlined in **Scheme** **2**. The corresponding monomers and PDPP3T were prepared according to the literature.^37^ Reaction of 1 with 1‐bromo‐2‐octyldodecane and diethyleneglycol 2‐bromoethyl methyl ether, followed by bromination with *N*‐bromosuccinimide, led to 2 in 10.2% yield. The Stille cross‐coupling of 2 with bis‐tin reagents 3 led to PDPP3T‐1. The co‐polymerization of compounds 3, 4, and 5 at a molar ratio of 2:1:1 yielded PDPP3T‐2. The resulting polymers were precipitated out from the reaction mixtures after addition of methanol, followed by Soxhlet extraction, giving PDPP3T‐1, PDPP3T‐2, and PDPP3T in 95.0%, 93.8%, and 92.0% yields, respectively. All chemical structures including the monomers and polymers were verified by ^1^H NMR and elemental analysis (Supporting Information). All polymers can be dissolved in *o*‐dichlorobenzene and 1,1,2,2‐tetrachloroethane. The *M*_w_ determined with high temperature gel permeation chromatography (GPC) of PDPP3T‐1, PDPP3T‐2, and PDPP3T was estimated to be 53.1, 44.0, and 68.5 kg mol^−1^ with polydispersities of 3.0, 4.1, and 2.2, respectively. The decomposition temperatures based on thermogravimetric analysis (TGA) curves (Figure S1, Supporting Information) of PDPP3T‐1 and PDPP3T‐2 were 391 and 386 °C, respectively, at 5% weight loss.

Cyclic voltammograms of PDPP3T‐1 and PDPP3T‐2 were measured in the form of thin films (Figure S2a, Supporting Information), along with the cyclic voltammogram of PDPP3T for comparison. All three polymers show one or two quasi‐reversible oxidation waves and one quasi‐reversible reduction wave. Based on the respective onset oxidation and reduction potentials, HOMO and LUMO energies of these conjugated polymers were estimated (Supporting Information).^38^ As listed in Table S1 (Supporting Information), the respective HOMO levels of PDPP3T‐1 (−5.13 eV) and PDPP3T‐2 (−5.10 eV) are higher than that of PDPP3T (−5.36 eV), and the respective LUMO levels of PDPP3T‐1 (−3.62 eV) and PDPP3T‐2 (−3.65 eV) are lower than that of PDPP3T (−3.44 eV). For comparison, the cyclic voltammograms of PDPP3T‐1, PDPP3T‐2, and PDPP3T in solutions were also measured (see Figure S2b, Supporting Information) and the respective HOMO and LUMO energies were deduced (see Table S2, Supporting Information). As listed in Table S2 (Supporting Information), the HOMO and LUMO levels of PDPP3T‐1 and PDPP3T‐2 estimated with the cyclic voltammograms of the polymer solutions are enhanced and lowered, respectively, in comparison with those of PDPP3T. Therefore, the alteration of HOMO/LUMO levels for PDPP3T‐1 and PDPP3T‐2 can be attributed to the partial replacement of branching alkyl chains with linear TEG chains, which is expected to reduce steric hindrances resulting from the branching alkyl chains, leading to more planar conjugated backbones. However, HOMO/LUMO levels of PDPP3T‐1 and PDPP3T‐2 estimated with the respective cyclic voltammograms in solutions are slightly different from those measured with the respective thin films. Thus, interchain π–π interactions may also contribute to the alteration of LUMO/HOMO levels of PDPP3T‐1 and PDPP3T‐2 in comparison with PDPP3T, as discussed below.

Figure S3 (Supporting Information) shows the absorption spectra of all three polymers in solutions and thin films, with all exhibiting broad absorptions extending to 1000 nm. Respective absorption maxima for solutions of PDPP3T‐1, PDPP3T‐2, and PDPP3T appear at 864, 848, and 818 nm, while each polymer exhibits an additional shoulder in the range of 760–780 nm. Thin films of PDPP3T‐1, PDPP3T‐2, and PDPP3T absorb strongly around 860, 850, and 832 nm, respectively. The absorption around 860 nm for the thin film of PDPP3T‐1 is almost overlapped with that of PDPP3T‐1 in solution, and moreover the absorption tail in the range of 870–1090 nm is redshifted for the thin film. The absorptions of PDPP3T‐1 and PDPP3T‐2 are redshifted in comparison with that of PDPP3T. Based on the respective absorption onset of their thin films, optical bandgaps were estimated to be 1.29, 1.30, and 1.36 eV for PDPP3T‐1, PDPP3T‐2, and PDPP3T, respectively. Thus, the bandgaps decrease in the following order: PDPP3T > PDPP3T‐2 > PDPP3T‐1. This is likely due to the fact that backbones of PDPP3T‐1 and PDPP3T‐2 are more planar than that of PDPP3T as discussed above. By comparing the respective solution and thin‐film absorptions, the shifts in maximum absorption between the solutions and thin films are much smaller for PDPP3T‐1 and PDPP3T‐2 than that for PDPP3T. This indicates that preaggregation of polymer chains of PDPP3T‐1 and PDPP3T‐2 may occur in solution, leading to greater orientational interchain ordering in thin films, according to previous studies.^39^, ^40^

**Figure 1:**
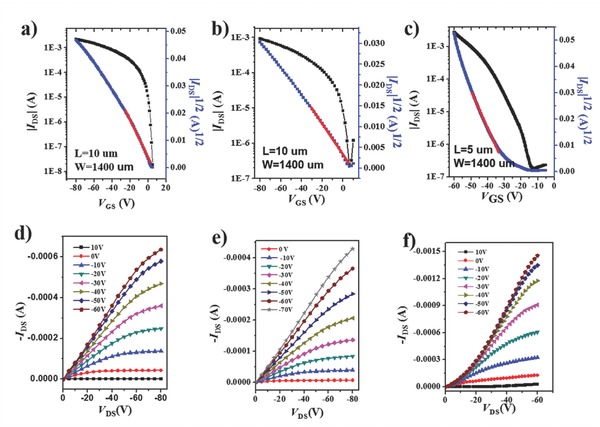
The transfer and output characteristics of a,d) as‐prepared PDPP3T‐1 based FETs, b,e) PDPP3T‐2, and c,f) PDPP3T based FETs after thermal annealing at 100 °C (*V*_DS_ for transfer characteristics is −80 V).

Bottom‐gate/bottom‐contact field effect transistors (FETs) were fabricated with commonly used methods and measured under ambient conditions to study the charge transport properties of these polymers. **Figure** **1**a–f shows the output and transfer characteristics of thin‐film FETs of the three polymers. We find that all exhibit typical *p*‐type behavior under ambient atmosphere. The FET performance data were extracted from the transfer curves and are listed in **Table** **1**. The as‐prepared thin film of PDPP3T‐1 exhibits high hole mobilities up to 2.6 cm^2^ V^−1^ s^−1^. In comparison, the as‐prepared thin films of both PDPP3T‐2 (0.11 cm^2^ V^−1^ s^−1^) and PDPP3T (0.39 cm^2^ V^−1^ s^−1^) possess comparatively low hole mobilities, although after thermal annealing at 100 °C hole mobilities of PDPP3T‐2 and PDPP3T increase to 0.65 and 1.48 cm^2^ V^−1^ s^−1^, respectively. Further thermal annealing at 150 °C, however, leads to a decrease in performance. Interestingly, the mobility of PDPP3T‐1 remains almost unaltered after thermal annealing at 100 °C, and only decreases to 1.7 cm^2^ V^−1^ s^−1^ after further annealing at 150 °C. This trend in charge mobility with annealing temperature is in strong contrast to that of most conjugated polymers (including PDPP3T‐2 and PDPP3T), where good charge transport is often realized only after thermal annealing.^4^, ^22^, ^41^ In the case of PDPP3T‐1 thermal annealing procedures are not required, allowing rapid device manufacturing and limiting thermal degradation. Additionally, it is worthwhile to note that FETs fabricated with PDPP3T‐1 display higher on/off ratios and relatively low threshold voltages (*V*_th_) compared to the other two polymers. These observations demonstrate that the incorporation of TEG chains in a regular arrangement as in PDPP3T‐1 is beneficial for charge transport. This conclusion is supported by the finding that a smaller enhancement in device performance is measured for PDPP3T‐2 in which the TEG chains are distributed along the conjugated backbone in a random manner.

**Table 1:** The FET performance parameters and π–π stacking distances of PDPP3T‐1, PDPP3T‐2, and PDPP3T

Polymer	Temperature [°C]	*µ*_h_ a) [cm^2^ V^−1^ s^−1^]	*I*_on/off_	*V*_th_/V	*d*_π–π_ [nm]
PDPP3T‐1	25	1.9 (2.6)	10^4^–10^5^	−5–6	
	100	2.0 (2.6)	10^4^–10^5^	−3–10	0.361
	150	0.8 (1.7)	10^4^–10^5^	−11–5	
PDPP3T‐2	25	0.06 (0.11)	10^3^–10^4^	−12–3	
	100	0.2 (0.65)	10^3^–10^4^	−25–10	0.365
	150	0.09 (0.12)	10^3^–10^4^	−20–9	
PDPP3T	25	0.22 (0.39)	10^3^–10^4^	−10–(−2)	
	100	0.9 (1.48)	10^3^–10^4^	−27–(−2)	0.373
	150	0.5 (1.0)	10^3^–10^4^	−12–(−3)	
PDPP3T^41^	150	1.57^max^			

^a)^ The mobilities were provided in “average(highest)” form, which were obtained based on more than ten devices with channel length (*L*) of either 5 or 10 µm.

**Figure 2:**
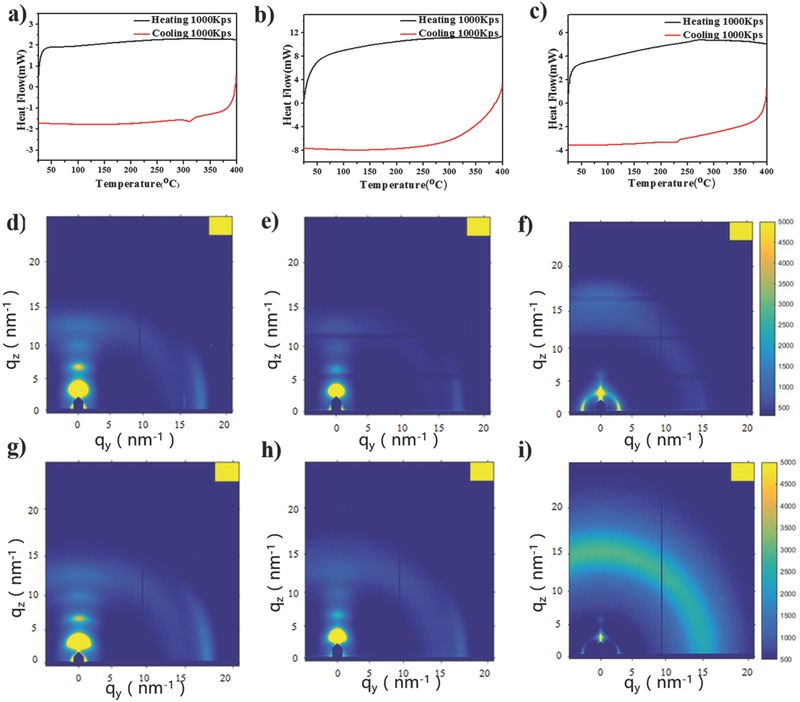
Flash DSC thermograms of a) PDPP3T‐1, b) PDPP3T‐2, and c) PDPP3T. GIWAXS patterns of the d–f) as‐prepared and g–i) thermally annealed thin‐films of d,g) PDPP3T‐1, e,h) PDPP3T‐2, and f,i) PDPP3T.

In order to understand the variation in charge mobilities between these three polymers with different side chain arrangements, their interchain packing order and thin‐film morphologies were characterized with ultrafast flash differential scanning calorimetry (F‐DSC), grazing incidence wide angle X‐ray scattering (GIWAXS), and atomic force microscopy. Because of the very fast heating/cooling rates, F‐DSC has been successfully utilized to investigate the phase behavior of organic functional materials, including even weak phase transitions that are not detectable in conventional DSC measurement.^42^ As shown in **Figure** **2**a–c, weak exotherms are observed only for PDPP3T‐1 and PDPP3T at ≈311 and 230 °C, respectively. We attribute these transitions to crystallization processes from the melt although they may also result from a solid/solid‐phase transition. In any case this observation indicates that both PDPP3T‐1 and PDPP3T can be molecularly ordered. The randomly substituted PDPP3T‐2 in contrast does not display any noticeable features, suggesting that this material has a low tendency to crystallize.

To obtain further structural insights, we performed GIWAXS. Figure 2d–i shows 2D GIWAXS patterns for thin films of PDPP3T‐1, PDPP3T‐2, and PDPP3T before and after annealing at 100 °C. The as‐prepared thin film of PDPP3T‐1 shows four orders of the out‐of‐plane diffractions that results from the lamellar packing of side chains at *q_z_* = 3.5, 7.0, 10.2, and 13.0 nm^−1^, corresponding to a d‐spacing of 1.79 nm. Moreover, a scattering signal at *q_y_* = 17.4 nm^−1^ in the in‐plane direction emerges for thin film of PDPP3T‐1, which is attributed to the interchain π−π stacking with a distance of 0.361 nm. These GIWAXS data indicate that polymer chains of PDPP3T‐1 are predominantly packed in edge‐on mode on the OTS (*n*‐octadecyltrichlorosilane) modified SiO_2_/Si substrate, which is believed to be favorable for charge transport.^2^, ^16^ It is noted that the GIWAXS pattern of PDPP3T‐1 is almost unchanged aside from a slight enhancement in scattering intensities after thermal annealing at 100 °C, in good agreement with the observation that thin‐film hole mobility remains almost unchanged following thermal annealing.

Thin films of PDPP3T‐2 display similar GIWAXS patterns as PDPP3T‐1. However, signal intensities for as‐cast PDPP3T‐2 are much weaker than those of PDPP3T‐1 (which remain weak even after thermal annealing), indicating a notably lower molecular order compared to PDPP3T‐1, in agreement with our thermal analysis data. It is important to highlight, though that edge‐on orientation of the macromolecules is still favored.

Compared to PDPP3T‐1 and PDPP3T‐2, the as‐prepared thin film of PDPP3T shows ring‐like scattering signals, with their intensities being enhanced after thermal annealing. The appearance of ring‐like signals at *q_z_* = *q_y_* = ≈16.8 nm^−1^ corresponds to a π−π stacking distance of 0.373 nm that is larger than those observed for PDPP3T‐1 and PDPP3T‐2 (Table 1). This may negatively affect charge transport. In addition, the ring‐like signal implies that a broad distribution of chain orientations is present; i.e., PDPP3T films display a notably lower anisotropy than both PDPP3T‐1 and PDPP3T‐2 for which a strong tendency for edge‐on orientation was found. We therefore conclude that the incorporation of TEG chains can improve the chain packing as well as induces a beneficial edge‐on chain orientation. The results also reveal that attaching TEG chains in a regular fashion as in PDPP3T‐1 is more favorable for improving molecular order than the random arrangement in PDPP3T‐2.

**Figure 3:**
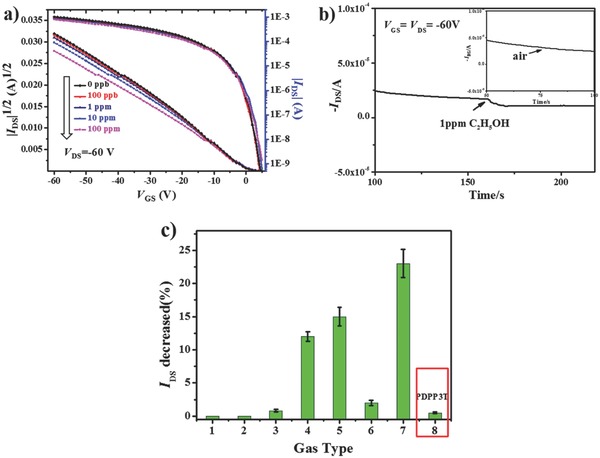
a) Transfer characteristics for PDPP3T‐1 FETs after exposure to different concentrations (0–100 ppm) of ethanol vapor. b) *I*_DS_ versus time under 1 ppm ethanol vapor and air (inset). c) Variation of the drain current for FET of PDPP3T‐1 after exposure to different gas vapors: 1, CO_2_ (pure); 2, CH_2_Cl_2_ (3 010 000 ppm); 3, hexane (20 000 ppm); 4, ethyl acetate (1000 ppm); 5, acetone (100 ppm); 6, acetone (1 ppm); 7, C_2_H_5_OH (1 ppm); and 8, C_2_H_5_OH (10 000 ppm) to PDPP3T under the same conditions.

The presence of TEG chains in PDPP3T‐1 is expected to endow the resulting FETs with additional functionalities. In the following, we demonstrate the sensitive and selective response of FETs with PDPP3T‐1 toward ethanol vapor. The gaseous analytes used in the experiments were prepared by controllable dilution with air (with a humidity of 15%) and their concentrations were presented in the v/v form. **Figure** **3**a shows the variation of the transfer characteristics for the FET with thin film of PDPP3T‐1 upon exposure to different concentrations of ethanol vapor. We observe that the on‐current (*I*_DS_) decreases after exposure to different ethanol vapor concentrations. For instance, the on‐current is reduced by 23% after exposure to 1.0 ppm of ethanol vapor (Figure 3b), with the reduction still detectable even when the concentration of ethanol vapor is down to 100 ppb. For comparison, the on‐current remains almost unaltered after exposure to air with a humidity of 15% (see Figure 3b).

The selectivity of PDPP3T‐1 FET toward ethanol vapor was investigated, with CO_2_ and vapors of different volatile solvents such as dichloromethane (CH_2_Cl_2_), hexane, ethyl acetate, and acetone chosen for a selectivity study. As depicted in Figure 3c, the variation of on‐current (*I*_DS_) can be neglected after exposure to CO_2_ and vapors of CH_2_Cl_2_ and hexane with low polarity even at high concentrations. Only when the concentrations of vapors of polar solvents such as ethyl acetate and acetone reached 1000 ppm and 100 ppm, respectively, the on‐current decrease could be detected, demonstrating that the PDPP3T‐1 FET shows good selectivity toward ethanol vapor. In comparison, the PDPP3T FET exhibits almost no response toward ethanol vapor, with negligible variation of on‐current even after exposure to 10 000 ppm of ethanol vapor. Thus, the sensitive and selective response of the FET with thin film of PDPP3T‐1 toward ethanol vapor is due to the polar TEG side chains.

## 3 Conclusion

In conclusion, we report two new DPP‐terthiophene‐based conjugated polymers with TEG side chains, i.e., PDPP3T‐1 and PDPP3T‐2. The TEG side chains are distributed uniformly in PDPP3T‐1, but arrange randomly in PDPP3T‐2, with their incorporation causing a significant effect on interchain packing. Thin films of PDPP3T‐1 show better molecular order and a beneficial edge‐on chain orientation compared to films of the PDPP3T polymer without TEG chains. This favorable microstructure of PDPP3T‐1 leads to a high hole mobility up to 2.6 cm^2^ V^−1^ s^−1^ in as‐cast films without the need for any post‐deposition treatments. The side chains also lead to other beneficial features. For instance, we demonstrated that by taking the advantage of the hydrophilic TEG chains in PDPP3T‐1, FETs made of this polymer can be successfully utilized for sensing ethanol vapor with high sensitivity down to 100 ppb and good selectivity. Together, these results clearly demonstrate that incorporation of functional chains into conjugated D‐A polymers can modify their interchain packing and thin‐film morphology, thus, enabling to tuning their semiconducting properties and assisting in creating multifunctional structures.

## Conflict of Interest

The authors declare no conflict of interest.

## Supporting information

Supplementary
