# Supplementary material for: Diketopyrrolopyrrole‐Based Conjugated Polymer Entailing Triethylene Glycols as Side Chains with High Thin‐Film Charge Mobility without Post‐Treatments
Source: Adv Sci (Weinh). 2017 Apr 18;4(8):1700048. doi: 10.1002/advs.201700048 (PMC5566237; doi:10.1002/advs.201700048)

Copyright WILEY-VCH Verlag GmbH & Co. KGaA, 69469 Weinheim, Germany, 2017.

ADVANCED SCIENCE Open Access

Supporting Information

for *Adv. Sci.,* DOI: 10.1002/advs.201700048

Diketopyrrolopyrrole-Based Conjugated Polymer Entailing Triethylene Glycols as Side Chains with High Thin-Film Charge Mobility without Post-Treatments

Si-Fen Yang, Zi-Tong Liu, Zheng-Xu Cai, Matthew J. Dyson, Natalie Stingelin, Wei Chen, Hua-Jun Ju, Guan-Xin Zhang, and De-Qing Zhang**

Copyright WILEY-VCH Verlag GmbH & Co. KGaA, 69469 Weinheim, Germany, 2016.

Supporting Information

Diketopyrrolopyrrole-based Conjugated Polymer Entailing Triethylene Glycols as Side Chains with High Thin Film Charge Mobility without Post-treatments

Si-Fen Yang, Zi-Tong Liu, Zheng-Xu Cai, Matthew J. Dyson, Natalie Stingelin, Wei Chen, Hua-Jun Ju, Guan-Xin Zhang, De-Qing Zhang**

Contents

1. **Materials and Characterization Techniques……………………………………….....…S2**
2. **Synthesis of Monomers and Polymers………………………….................................S3**
3. **TGA Curves of PDPP3T-1, PDPP3T-2, and PDPP3T………………………………..S5**
4. **Cyclic Voltammograms of PDPP3T-1, PDPP3T-2 and PDPP3T…………….....……S5**
5. **Absorption Maxima, Redox Potentials, HOMO/LUMO Energies and Bandgaps of PDPP3T-1, PDPP3T-2 and PDPP3T………………………………………………..……..S6**
6. **AFM Images………………………………………………………………….…..........S7**
7. **Fabrication of Bottom-gate/bottom-contact OFET Devices……………………..........S7**
8. **NMR Spectra of Monomers and Polymers………………….......................….….........S9**

1. Materials and Characterization Techniques

Materials: If not specified elsewhere, the starting materials and reagents were commercially available and used directly. Compounds **4** and **5** (Scheme 2) were prepared by following the reported procedures.[S1]

Characterization Techniques: ^{1}H NMR and ^{13}C NMR spectra were measured with Bruker AVANCE III 300 MHz and 400 MHz spectrometers. Matix-Assisted Laser Desorption/Ionization TOF were performed on BEFLEX III spectrometer. Elemental analysis were recorded on a Carlo Erba model 1160 elemental analyzer. TGA (Shimadzu DTG-60) measurements were performed on Shimadzu DTG-60 at a heating rate of 10 K/min under a nitrogen atmosphere. GPC analysis was carried out on an PL-GPC 220 high temperature chromatograph equipped with a IR5 detector at 150 ^oC; polystyrene was utilized as the calibration standard and 1,2,4-trichlorobenzene as eluent (1.0 mL/min). Cyclic voltammograms were measured with a conventional three-electrode cell, in which Ag/AgCl as the reference electrode and Pt wires of 2 mm diameter as working and counter electrodes on a computer-controlled CHI660C instrument at ambient temperature; n-Bu_4NPF_6 was used as the conducting electrolyte. The onset oxidation and reduction potentials were presented by reference to the redox potential of ferrocene/ferrocenium (Fc/Fc^+); HOMO and LUMO energies of these conjugated polymers were estimated with the following equations: HOMO = $-(E_{ox}^{onset} + 4.8)$ eV, LUMO = $-(E_{red}^{onset} + 4.8)$ eV. JASCO V-570 UV-vis spectrophotometer was used to record the absorption spectra of the conjugated polymers (solutions and thin films). Atomic-force microscopy images of thin-films of polymer were taken by using a Digital Instruments Nanoscope V atomic force microscope operated in tapping mode with a Nanoscope V instrument in air.

Flash DSC was performed with a Mettler-Toledo Flash DSC 1 under N_2 atmosphere. The UFS1 differential scanning calorimeter chips (i.e., a thin film chip sensor composed of a thin free standing SiNx film on a silicon frame with a measuring area of 60 μm × 60 μm) were calibrated by the manufacturer and conditioned prior to use. Powder samples were then placed directly in contact with the sensor. Before data were recorded, the samples were heated to 400 °C and back to -80 °C at a rate of 1000 K s^{-1}, which erased the thermal history and ensured sufficient contact between the sample and sensor.

GIWAXS measurements were performed at the 8ID-E beamline at the Advanced Photon Source (APS), Argonne National Laboratory, using X-rays with a wavelength of λ = 1.6868 Å and a beam size of ~200 μm (h) and 20 μm (v). To make the results comparable to those of FET devices, the samples for the measurements were prepared on OTS modified SiO_2

substrates under the same conditions as those used for fabrication of OFET devices. A 2-D PILATUS 1M-F detector was used to capture the scattering patterns and was situated at 208.7 mm from samples. Typical GIWAXS patterns were taken at an incidence angle of 0.20°, above the critical angles of neat polymers and below the critical angle of while the q_z linecut was achieved by a linecut at $q_y = 0$ nm^{-1} using the reflected beam center as zero the substrate. Consequently, the entire structure of thin films could be detected. In addition, the q_y linecut was obtained from a linecut across the reflection beam center. The background of these linecuts was estimated by fitting an exponential function, and the parameters of the scattering peaks were obtained through the best fitting using the Pseudo-Voigt type 1 peak function.

[S1] C. Kanimozhi, N. Yaacobi-Gross, K. W. Chou, A. Amassian, T. D. Anthopoulos, S. Patil, *J. Am. Chem. Soc.* **2012**, *134*, 16532.

2. Synthesis of Monomers and Polymers

Synthesis of Compound 2. Compound **1** (5.0 g, 16.7 mmol), K_2CO_3 (5.0 g, 36.2 mmol) and DMF (150 mL) were added to a 250 mL double-neck round-bottom flask. The reaction container was heated to 120 °C for 30 min. 1-Bromo-2-octyldodecane (6.0 g, 16.7 mmol) and diethylene glycol 2-bromoethyl methyl ether (3.79 g, 16.7 mmol) were slowly added dropwise for 2 hrs, and the reaction mixture was stirred for 36 h at 130 °C. Solvents were removed by rotary evaporation and the residue was roughly purified by column chromatography. 3.0 g of Purple-brown crude product was obtained without further purification.

In a 250 mL round-bottom flask, 1.0 g of the purple-brown solid was dissolved in 150 mL of $CHCl_3$ under nitrogen atmosphere. *N*-Bromosuccinimide (0.62 g, 3.48 mmol) was added, and the reaction mixture was stirred for 4 h at room temperature. After that, the reaction mixture was poured into water, and the organic phase was separated and washed by water. The organic layers were dried over $MgSO_4$ and concentrated by rotary evaporation. The crude compound was purified by column chromatography with CH_2Cl_2 and ethyl acetate (5:1, v/v) as the eluent. Compound **2** was obtained as a dark purple solid (500 mg, 10.2%). ^{1}H NMR (300 MHz, $CDCl_3$, δ): 8.58 (d, *J* = 6 Hz, 1H), 8.53 (d, *J* = 3 Hz, 1H), 7.23-7.20 (m, 2H), 4.19-4.17 (m, 2H), 3.93-3.90 (m, 2H), 3.80-3.76 (m, 2H), 3.65-3.47 (m, 8H), 3.35 (s, 3H), 1.87 (s, 1H), 1.23 (m, 32H), 0.88-0.86 (m, 6H); ^{13}C NMR (100 MHz, $CDCl_3$, δ): 161.5, 161.2, 139.6, 139.3, 135.2, 134.9, 131.4, 131.2, 119.2, 119.1, 108.2, 107.9, 72.0, 70.8, 70.6, 69.0, 59.0, 46.3, 42.3, 37.8, 31.9, 31.2, 30.0, 29.6, 29.5, 29.4, 29.3, 26.2, 22.7, 14.1; MAILD-TOF *m/z*: 907.2 [M+Na$^+$]. Anal. calcd for $C_{41}H_{60}Br_2N_2O_5S_2$: C, 55.65; H, 6.83; N, 3.17; S, 7.25; found: C, 55.62; H, 6.88; N, 3.10; S, 7.12.

Synthesis of **PDPP3T-1**. Compounds **2** (150 mg, 0.17 mmol) and **3** (69.5 mg, 0.17 mmol)

were taken into a Schlenk tube under nitrogen atmosphere with 10 mL of anhydrous toluene. Then, tris(dibenzylideneacetone)dipalladium (0) ($Pd_2(dba)_3$) (1.56 mg) and tri(*o*-tolyl)phosphine (P(*o*-tol)$_3$) (4.1 mg) were added in one portion. The tube was charged with nitrogen through a freeze-pump-thaw cycle for three times. The mixture was stirred for 36 h at 100 ^oC under nitrogen. After cooling to room temperature, the highly viscous black gel-like solution was poured into methanol and the resulting precipitate was filtered. The polymer was purified by Soxhlet extraction using methanol, acetone, hexane and chloroform sequentially. The residue was collected and dried under vacuum to afford polymer (130 mg, 95.0%). ^1H NMR (400 MHz, $CDCl_2CDCl_2$, 100^oC, δ): 8.93 (m, br, 2H), 7.15 (m, br, 4H), 4.29-3.56 (m, br, 14H), 3.39 (m, br, 3H), 2.06 (m, br, 1H), 1.47-1.34 (m, br, 32H), 0.93 (m, br, 6H). GPC: M_n = 17.6 kDa, M_w = 53.1 kDa, PDI = 3.0. Anal. calcd for $(C_{45}H_{62}N_2O_5S_3)_n$: C, 66.96; H, 7.74; N, 3.47; S, 11.92; found: C, 66.05; H, 7.73; N, 3.36; S, 11.65.

***Synthesis of* PDPP3T-2.** Compounds **4** (50.0 mg, 0.067 mmol), **5** (68.0 mg, 0.067 mmol) and **3** (54.6 mg, 0.133 mmol) were taken in a Schlenk tube under nitrogen atmosphere with 10 mL of anhydrous toluene, followed by the addition of tris(dibenzylideneacetone)dipalladium (0) ($Pd_2(dba)_3$) (0.61 mg) and tri(*o*-tolyl)phosphine (P(*o*-tol)$_3$) (1.62 mg) in one portion. The tube was charged with nitrogen through a freeze-pump-thaw cycle for three times. The mixture was stirred for 36 h at 100 ^oC under nitrogen. After cooling to room temperature, the highly viscous black gel-like solution was poured into methanol and the resulting precipitate was filtered. The polymer was purified by Soxhlet extraction using methanol, acetone, hexane and chloroform sequentially. The residue was collected and dried under vacuum to afford polymer (100 mg, 93.8%). ^1H NMR (400 MHz, $CDCl_2CDCl_2$, 100^oC, δ): 8.87 (m, br, 2H), 7.15 (m, br, 4H), 4.35-3.55 (m, br, 14H), 3.38 (m, br, 3H), 2.04 (m, br, 1H), 1.47-1.31 (m, br, 32H), 0.93 (m, br, 6H). GPC: M_n = 10.6 kDa, M_w = 44.0 kDa, PDI = 4.1. Anal. calcd for $(C_{89}H_{122}N_4O_{10}S_6)_n$: C, 66.80; H, 7.68; N, 3.50, S, 12.02; found: C, 66.60; H, 7.58; N, 3.79; S, 12.22.

***Synthesis of* PDPP3T. PDPP3T** were prepared by following the reported procedures.[S2] ^1H NMR (400 MHz, $CDCl_2CDCl_2$, 100^oC, δ): 8.80 (m, br, 2H), 7.35-7.01 (m, br, 4H), 4.04 (m, br, 4H), 1.99 (m, br, 2H), 1.42-1.26 (m, br, 64H), 0.87 (m, br, 12H); GPC: M_n = 30.9 kDa, M_w = 68.5 kDa, PDI = 2.2. Anal. calcd. for $(C_{58}H_{88}N_2O_2S_3)_n$: C, 73.99; H, 9.42; N, 2.98; S, 10.22; Found: C, 74.11; H, 9.57; N, 2.82; S, 10.42.

[S2] J. C. Bijleveld, A. P. Zoombelt, S. G. J. Mathijssen, M. M. Wienk, M. Turbiez, D. M. de Leeuw, R. A. J. Janssen, *J. Am. Chem. Soc.* **2009**, *131*, 16616.

3. TGA Curves of PDPP3T-1, PDPP3T-2 and PDPP3T.

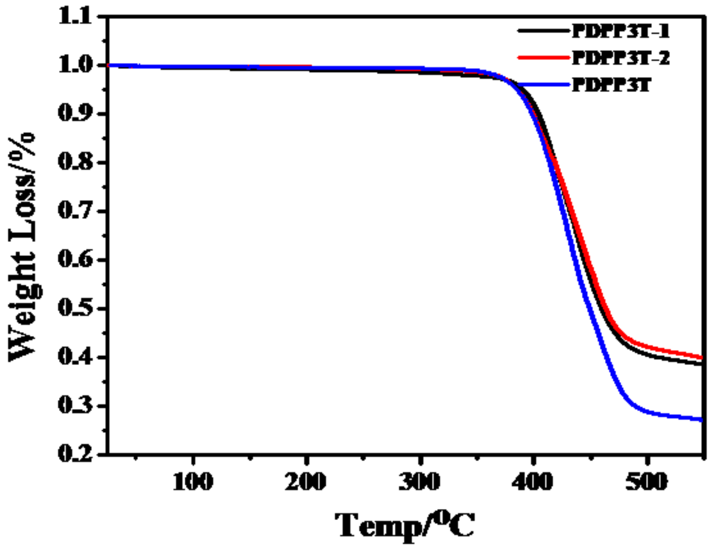

Figure S1. TGA curves of **PDPP3T-1**, **PDPP3T-2** and **PDPP3T**; heating rate: 10 K/min. from 20 °C to 550 °C under nitrogen atmosphere.

4. Cyclic Voltammograms of PDPP3T-1, PDPP3T-2 and PDPP3T.

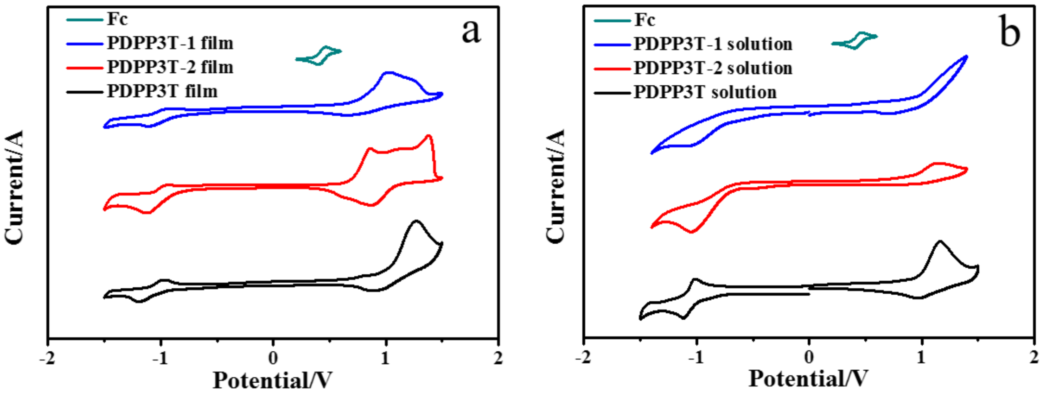

Figure S2 Cyclic voltammograms of thin films and solutions (1.0×10^{-3} M in hot 1,1,2,2-tetrachloroethane (100 °C)) of **PDPP3T-1**, **PDPP3T-2** and **PDPP3T**.

5. **Absorption Maxima, Redox Potentials, HOMO/LUMO Energies and Bandgaps** of **PDPP3T-1**, **PDPP3T-2** and **PDPP3T**.

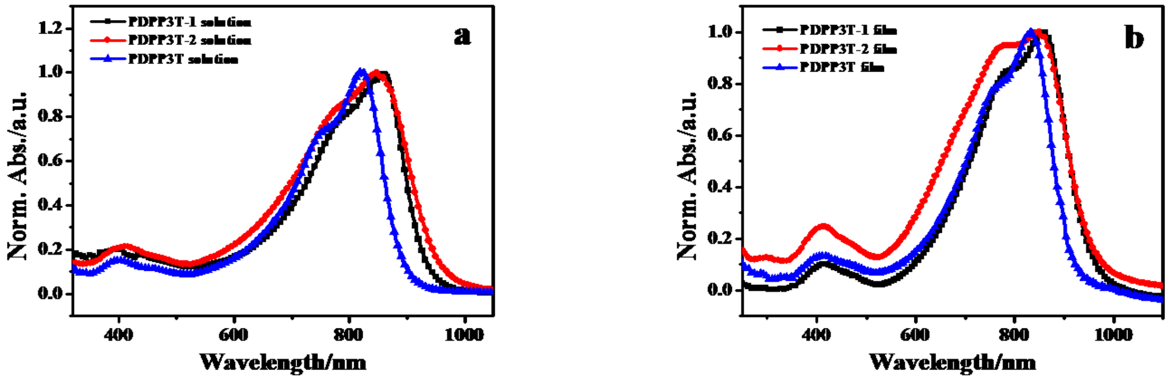

Figure S3. Normalized absorption spectra of **DPP3T-1**, **PDPP3T-2** and **PDPP3T** in 1,2-dichlorobenzene solution (1.0 × 10^{-5} M) and their thin films on a quartz plate

Table S1. Absorption maxima, redox potentials, HOMO/LUMO energies and bandgaps of **PDPP3T-1**, **PDPP3T-2** and **PDPP3T**

Polymer	$\lambda_{max}{}^{a}$ (nm) solution	$\lambda_{max}{}^{a}$ (nm) film	$E_{ox}{}^{onset}$ (V) [b]	$E_{red}{}^{onset}$ (V) [b]	HOMO (eV)	LUMO (eV)	$E_{g,cv}$ (eV) [c]	$E_{g,opt}$ (eV) [d]
**PDPP3T-1**	864	780, 860	0.33	-1.18	-5.13	-3.62	1.51	1.29
**PDPP3T-2**	848	770, 850	0.30	-1.15	-5.10	-3.65	1.45	1.30
**PDPP3T**	818	759, 832	0.56	-1.36	-5.36	-3.44	1.92	1.36

[a]Absorption maxima in 1,2-dichlorobenzene solution (1.0×10^{-5} M for each polymer) and the spin-coated thin film; [b]Onset potentials (V vs Fc/Fc^{+}) for reduction ($E_{redl}{}^{onset}$) and oxidation ($E_{oxl}{}^{onset}$); [c]Based on redox potentials; [d]Based on the absorption spectral data.

Table S2. Redox potentials and HOMO/LUMO energies of **PDPP3T-1**, **PDPP3T-2** and **PDPP3T** determined by solutions

Polymer	$E_{ox}{}^{onset}$ (V) [a]	$E_{red}{}^{onset}$ (V) [a]	HOMO (eV)	LUMO (eV)
**PDPP3T-1**	0.53	-1.17	-5.33	-3.63
**PDPP3T-2**	0.49	-1.15	-5.29	-3.65
**PDPP3T**	0.61	-1.34	-5.41	-3.46

[a]The onset oxidation and reduction potentials were presented by reference to the redox potential of ferrocene/ferrocenium (Fc/Fc^{+}); HOMO and LUMO energies of these conjugated polymers were estimated with the following equations: HOMO = -($E_{ox}{}^{onset}$ + 4.8) eV, LUMO = -($E_{red}{}^{onset}$ + 4.8) eV.

6. AFM Images.

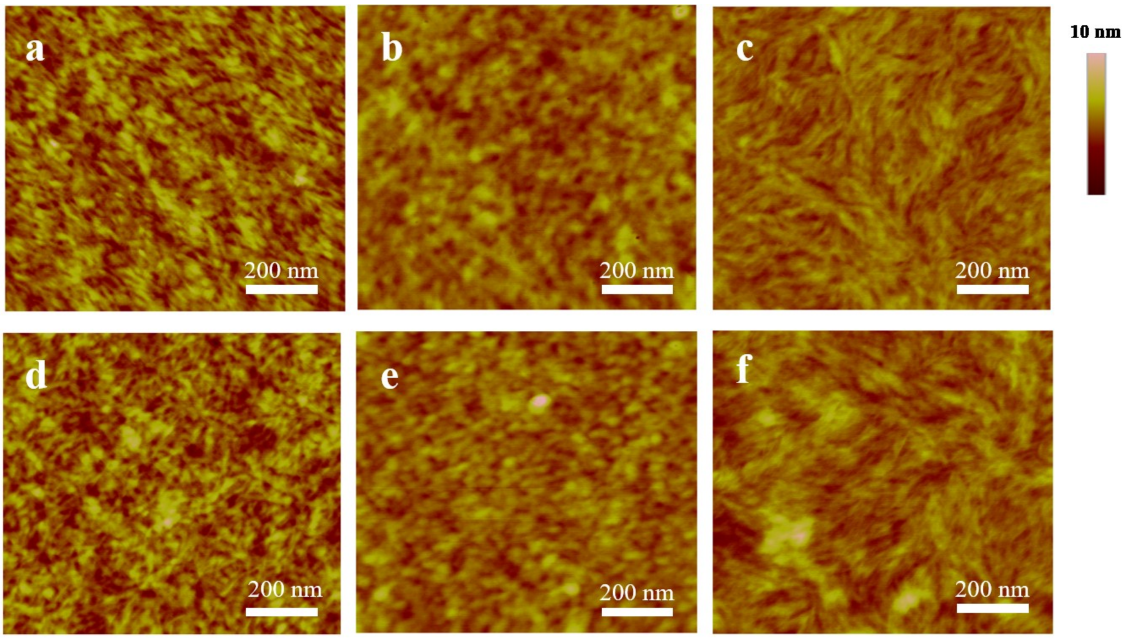

Figure S4. AFM images of the as-prepared (*up*) and thermally annealed at 100°C (*down*) thin-films of **PDPP3T-1** (a, d), **PDPP3T-2** (b, e) and **PDPP3T** (c, f).

Incorporating of TEG chains also affects the thin film morphology. AFM images of thin films of PDPP3T-1 and PDPP3T-2 before and after thermal annealing (Figure S4) are obviously different from those of PDPP3T, with the surfaces of the latter are more uniform with lower root-mean-square roughness. The AFM images indicate that the as-prepared thin film of PDPP3T contains interconnected thin fibers which further aggregate into large domains after thermal annealing. In contrast, the as-prepared thin film of PDPP3T-1 has cross-connected short rods, with thin film morphology almost unaltered after thermal annealing, based on the AFM images. Thin films of PDPP3T-2 comprise short rods and scattered particles before and after thermal annealing. Boundary areas exist among short rods and particles, and the existence of such boundary areas is expected to be detrimental for charge transport, in agreement with the observation that thin film of PDPP3T shows low charge mobility.

7. Fabrication of Bottom-gate/bottom-contact FET Devices.

A heavily doped *n*-type Si wafer was used as a gate electrode, while a layer of dry oxidized SiO_2 (300 nm, with roughness lower than 0.1 nm and capacitance of 11 nF cm^{-2}) was employed as the gate dielectric layer. The drain-source (*D-S*) gold contacts were fabricated by photo-lithography. The substrates were first cleaned by sonication in acetone and water for 5.0 min., and immersed in Piranha solution (2: 1 mixture of sulfuric acid and 30% hydrogen peroxide) for 20 min. The substrates were further rinsed with deionized water and isopropyl

alcohol for several times. After the substrate surfaces were modified with *n*-octadecyltrichlorosilane (OTS), they were washed with $CHCl_3$, *n*-hexane and isopropyl alcohol sequentially. Thin films were prepared by spin-coating their hot *o*-1,2-dichlorobenzene solutions (3.0 mg/mL) onto the modified substrates at 2000 rpm for 60 s. The annealing process was performed in vacuum for 2.0 hr at each temperature. The transfer and output curves of FETs were measured under ambient atmosphere using a Keithley 4200 SCS semiconductor parameter analyzer.

The charge mobility of the OFETs in the saturation region was obtained based on the following equation:

$$I_{DS} = \frac{W}{2L}\mu C_i \left(V_{GS} - V_{th}\right)^2$$

Where I_{DS} is the drain electrode collected current; L and W are the channel length and width, respectively; μ is the charge mobility of the device; C_i is the capacitance per unit area of the gate dielectric layer; V_{GS} and V_{th} are the gate and the threshold voltages, respectively. The V_{th} of the device was obtained by extrapolating the $(I_{DS,sat})^{1/2}$ vs. V_{GS} plot to $I_{DS} = 0$.

8. NMR Spectra of Monomers and Polymers

^{1}H NMR and ^{13}CNMR *of Compound 2*

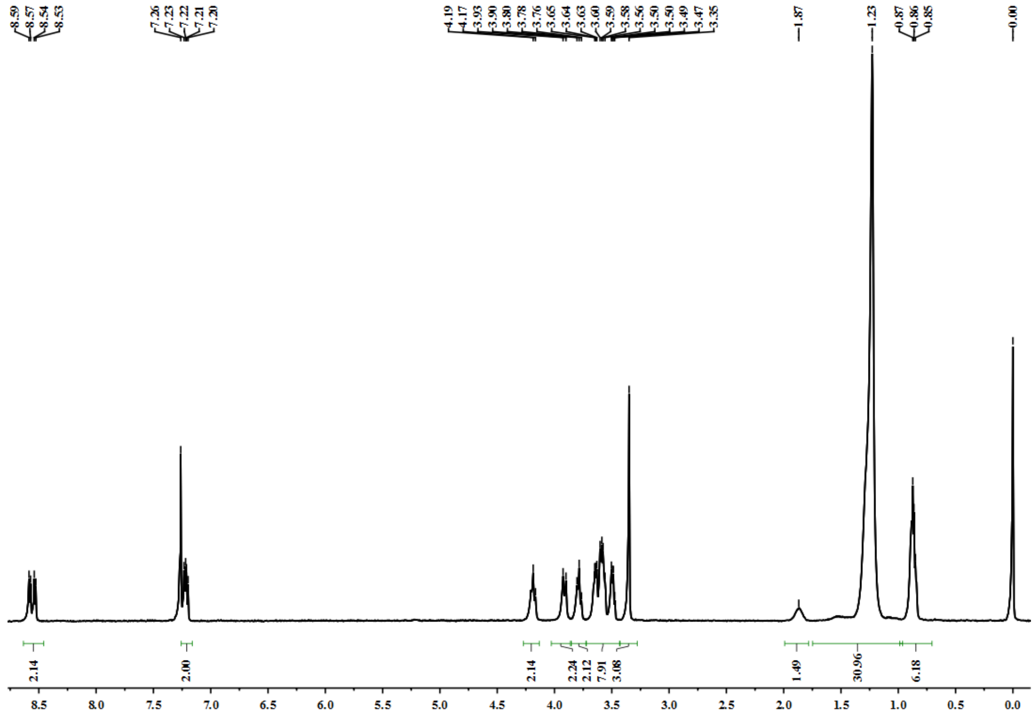

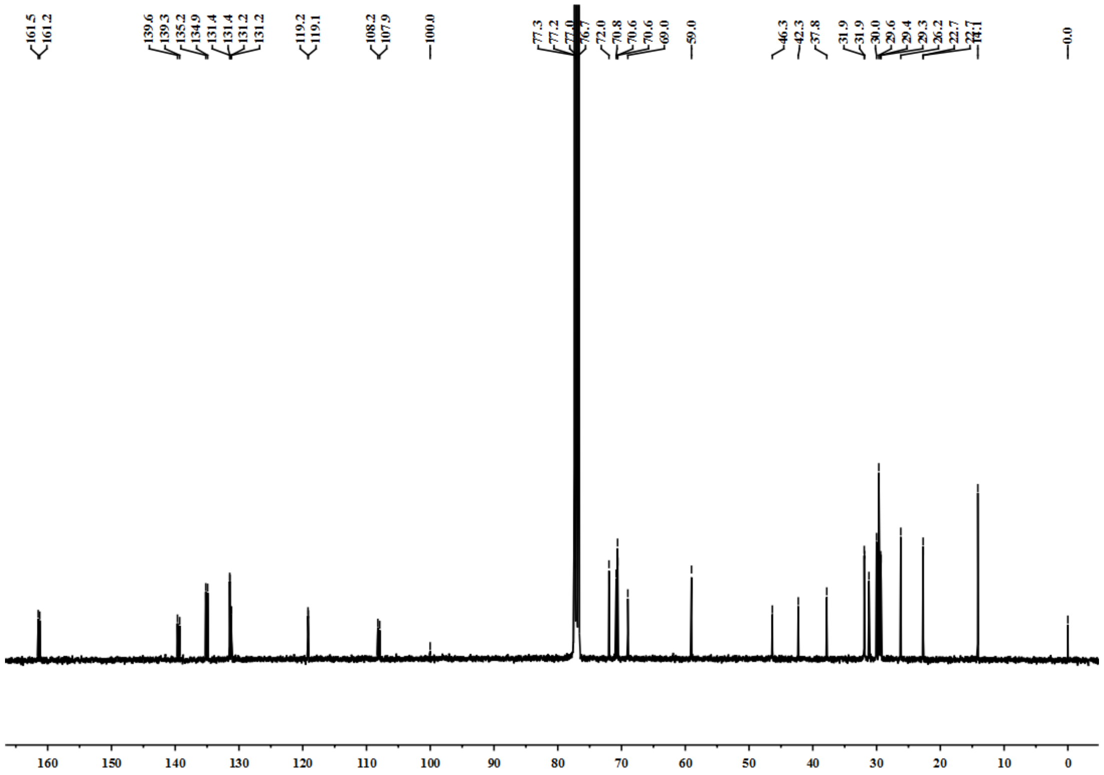

^{1}H NMR of PDPP3T-1

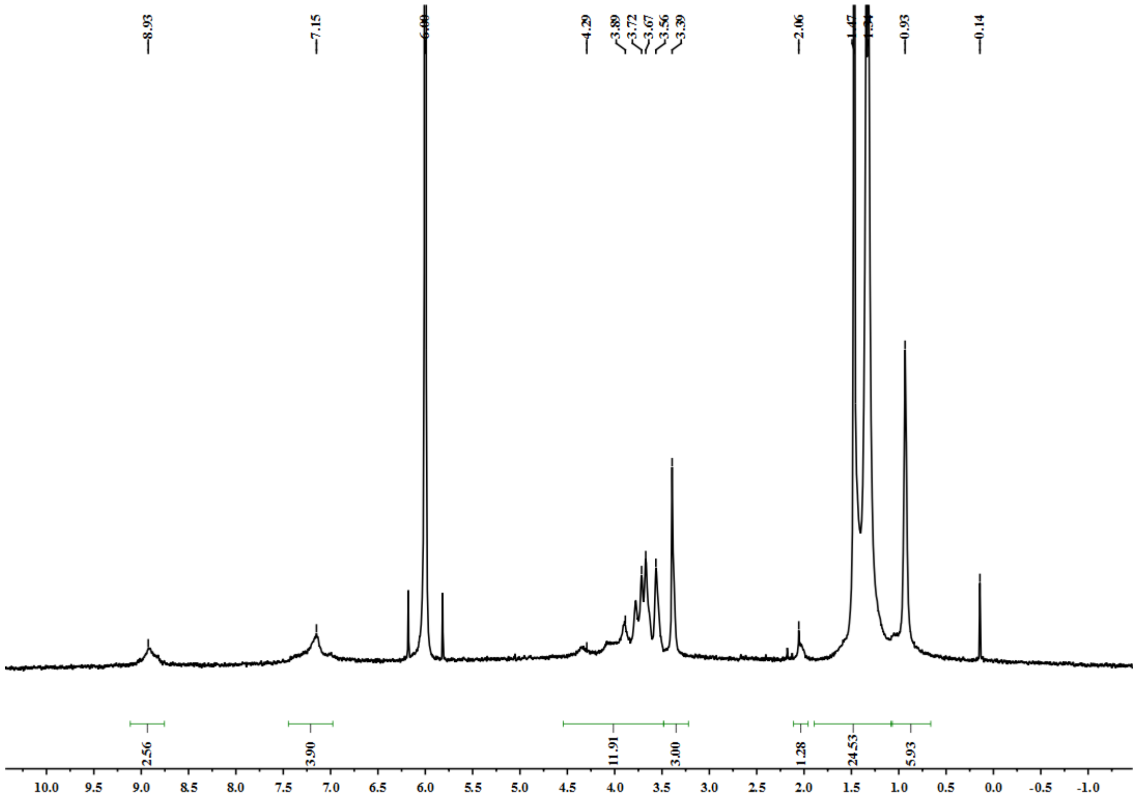

^{1}H NMR of PDPP3T-2

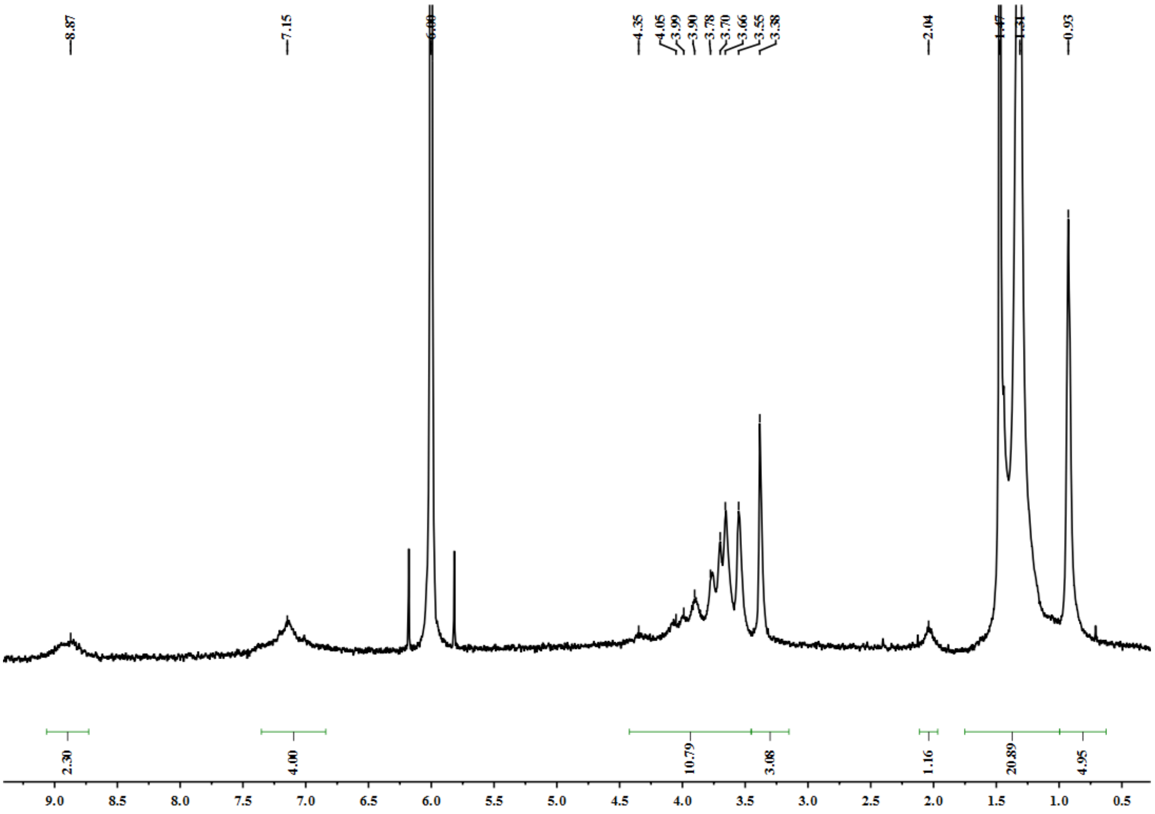

^{1}H NMR of PDPP3T

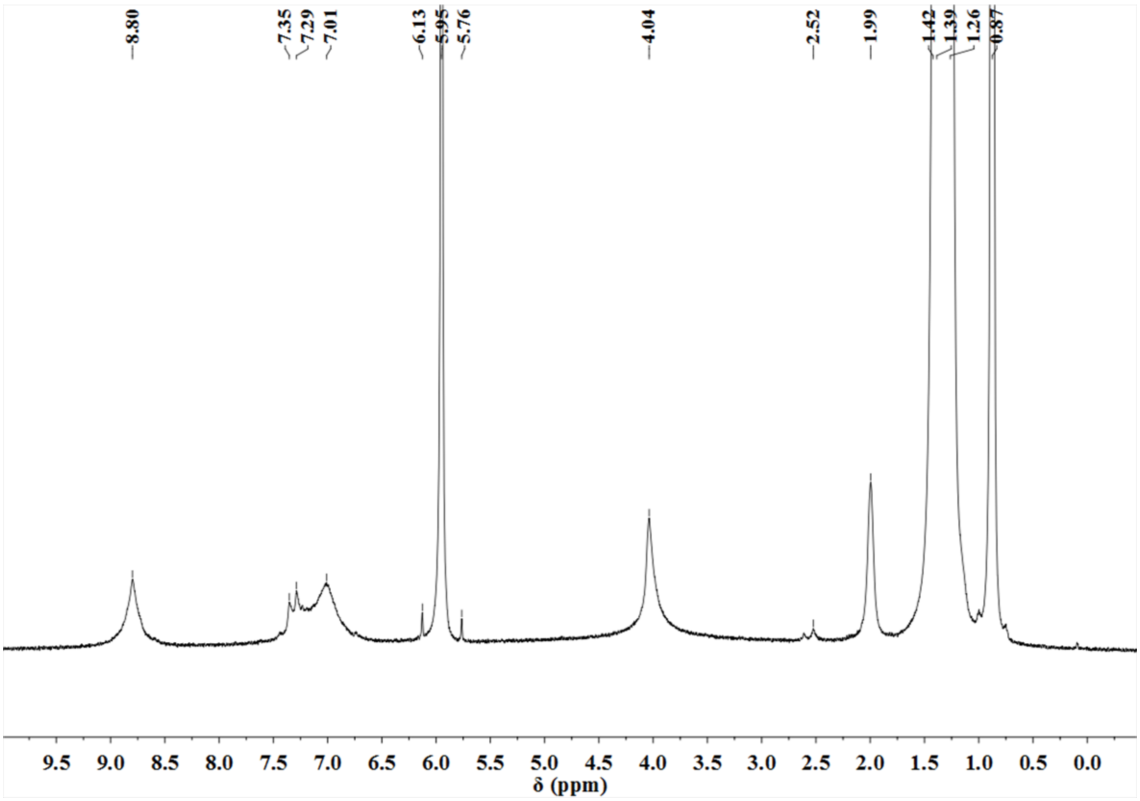

Supplement: Supplementary file 1 — Supplementary [file ADVS-4-na-s001.pdf]
